# Comparison of Maraging Steel Surface Integrity in Hybrid and Conventional Micro-ECDM Processes

**DOI:** 10.3390/ma15134378

**Published:** 2022-06-21

**Authors:** Niladri Mandal, Sergej Hloch, Alok Kumar Das

**Affiliations:** 1 Department of Mechanical Engineering, Indian Institute of Technology (ISM), Dhanbad 826004, India; alokmech@iitism.ac.in; 2 Faculty of Manufacturing Technologies, Technical University of Kosice with a Seat in Prešov, Štúrova 31, 08001 Prešov, Slovakia

**Keywords:** micro-ECDM, roughness, MRR, microstructure, ANOVA, micro-fabrication, powder, electrolyte

## Abstract

Maraging steel is one of the exotic materials showing the potential for application in the field of the aerospace industry. However, machining these materials with high surface quality and material removal rate is problematic. The micro-electro chemical discharge (MECDM) process is capable of resolving this problem to some extent, however, due to the spark action, it fails to attain a high surface finish. In the current investigation, micro-hole drilling is performed on maraging steel with powder-mixed alumina (1% wt. of Al_2_O_3_) using the micro-electro chemical discharge machining (PMECDM) process. The effect of different input process factors, for example, voltage (V), duty cycle (D), the electrolyte concentration (C), are considered for investigating the machining performance, i.e., rate of material removal (MRR) and roughness of surface (SR) of the machined substrate. Further, a comparative analysis is established between micro-ECDM (MECDM) and mixed powder ECDM (PMECDM). The Box–Behnken design is used to conduct all the experiments and analysis of variance (ANOVA) is used to optimize the results. The outcomes reveal that MRR in PMECDM is enhanced by 34%, and the average surface roughness is reduced by 21% over the MECDM process. The maximum MRR was observed to be 2.44 mg/min and the hole machined by the PMECDM results in a cleaner hole wall surface than the MECDM process due to the grinding action by the powder particles. The residual stress measurement indicates that the PMECDM (−128.3 ± 3.85 MPa) has the lowest equivalent stress as compared to the parent material (−341.04 ± 10.24 MPa) and MECDM (−200.7 ± 6.02 MPa) surfaces. The applied voltage is the most significant parameter, followed by the duty factor and electrolyte concentration for enhancing the MRR and surface finish. The addition of powder improves the surface integrity of the machined surface as compared to the surfaces produced by the MECDM processes.

## 1. Introduction

Maraging steel is one of the advanced aerospace materials with special mechanical properties such as ultra-high strength, hardness and fracture toughness. It shows an enormous potential application in the field of gas turbines and jet engines, where high-temperature sustainability is required such as in exhaust nozzles [1]. However, traditional and non-conventional machining processes struggle to achieve good surface quality and machining rate during the machining of this exotic material.

The micro-electro chemical discharge machining process is emerging as a promising micromachining process for developing 2D and 3D micro features in electrically conductive and non-conductive materials. It is frequently used in machining brittle and difficult-to-cut materials, such as glass, quartz, ceramic [2] and metal matrix composites [3].

**Figure 1:**
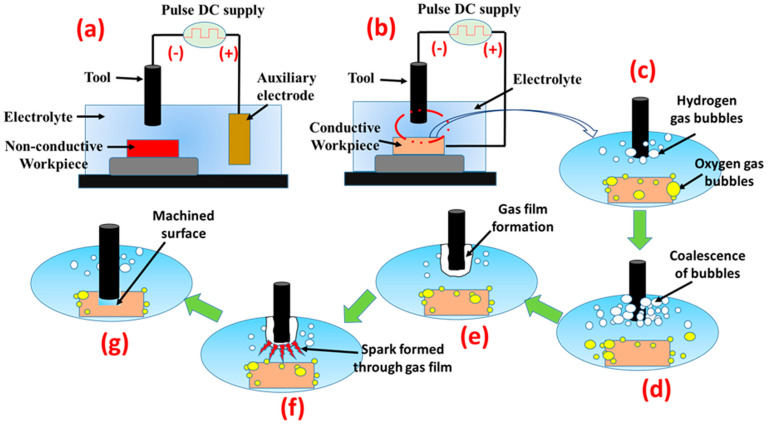
Working principle of μECDM for conductive and non-conductive materials [7]. (**a**) non-conductive material (**b**) conductive material (**c**–**f**) electrolysis process and spark generation (**g**) machined hole.

In this method, the combined action of thermal erosion followed by vaporization (by micro-EDM) [4] and electro-chemical etching (by micro-ECM) is responsible for the erosion of materials from the workpiece [5]. Due to the hybridization of the process, a higher rate of material removal (MRR) is attained compared to parent machining processes [6]. Figure 1 explains the principle and steps involved in the µ-ECDM process. Figure 1a,b represent the setup configuration for non-conductive and conductive material machining, respectively. In both these cases, the hydrogen gas bubbles evolved at the tool (diameter <1 mm) surface. An auxiliary electrode is used as an anode while machining non-conductive materials (Figure 1a) to establish an electro-chemical reaction. For the processing of the conductive workpiece, no such electrode is required (Figure 1b) as the reaction takes place between the workpiece (acting as the anode) and the tool. Pulsed DC supply is connected across the cathode and anode. During pulse-on-time, material removal occurs due to the electro-chemical reaction and sparking phenomenon.

On the other hand, during pulse-off-time, no spark happens due to which the debris is flushed out from the narrow inter-electrode gap. Therefore, multiple sparks promote the material removal from the surface. Figure 1c–e represent the steps involved in generating hydrogen gas bubbles followed by the formation of the passivation layer. This leads to the seizure of electro-chemical reaction; on increasing the applied voltage, this layer breaks down, and a spark is established (Figure 1f). The material placed in the close vicinity of these sparks melts and evaporates because of the heat energy of the sparks (Figure 1g). Unlike the EDM process, the recast layer is significantly minimized due to the electro-chemical reactions at high temperatures [7]. However, due to the electric discharge phenomenon, small craters and micro-cracks develop over the wall of the micro-hole, which causes roughness on the machined surface or even failure of the finished goods [8]. In addition, it degenerates the mechanical capabilities of the components [9].

In order to overcome this limitation of the conventional micro-ECDM process, hybridization of the process is required. Recently, researchers around the globe have been struggling to improve the efficiency of this procedure to reach a high level of geometrical accuracy and surface quality. The integration of ultrasonic vibration [10] and a magnetic field [11] with the micro-ECDM process are some of the hybridization methods adopted so far [12]. Another researcher applied a machine vision approach to understand the state of the ECDM-drilled hole in quartz glass and try to improve the machining performance [13]. Further, a mathematical model was developed by another investigator to understand or guide the machining operation [14]. Several approaches were stated earlier in this work to enhance the machining speed, dimensional accuracy, surface quality and removal rate of material [2]. However, despite numerous advantages, these hybrid processes require special attachments/approaches, making the machining operation expensive and complex [10]. Moreover, the ultrasonic vibration may rupture the gas film and promote the electro-chemical reaction, which is detrimental to the formation of sparks. 

Hybridization of the µECDM with a powder-added electrolyte is the most attractive and simple alternative to enhance machining performance. In this process, the machine configuration need not be altered. Nano or micron-sized ceramic powder is mixed in the electrolyte to improve the debris cleaning ability of the process by creating a grinding effect on the surface and providing a smooth surface compared to the micro-ECDM process. In the literature, Han et al. [15] added µ-sized graphite powder with a NaOH solution and investigated the relationship between the discharge waveform and surface quality. It was observed that the spark energy was reduced while using conductive particles due to a reduction in critical breakdown strength. Dispersion of the discharge energy was also observed, which improved the surface finish. They further concluded that the surface integrity was enhanced due to the absence of cracks on the machined surface and the reduction of average SR went from 4.86 µm to 1.44 µm. Yang et al. [16] added SiC abrasive powder to improve the quality of the slit machined by the wire-ECDM process. It was reported that the surface roughness could be further reduced by increasing the electrolyte concentration using smaller-sized abrasive particles. Kuo et al. [17] used a micron-sized SiC powder-added electrolyte to boost the machining performance of WECDM. It was observed that the modified electrolyte removes the debris efficiently due to the enhancement of electrolyte circulation. They obtained an 80% improvement in the surface finish of micro slits and concluded that titrated electrolytes make the process cost-effective and environmentally friendly. Elhami et al. [18] performed µ-drilling of glass using a copper tool and Al_2_O_3_ nanopowder-mixed electrolyte. They observed an increase in many sparks, which led to an increase in the material removal rate and overcut.

Few investigations have also been performed on conductive materials such as high-speed steel, stainless steel [19], copper, tantalum [20], and ceramic-coated Ni-super alloy with the conventional ECDM process [9]. Most of the reported work focused on dimensional accuracy and axial tool wear rate that occurred during these micro-drilling operations. Huang et al. [19] observed that the machining accuracy of a micro-hole drilled on ANSI 304 stainless steel was improved by enhancing the tool rotation speed. Coteaţă et al. [21] performed through-hole machining on an HSS sheet of 1.5 mm thickness in a sodium silicate electrolyte solution. Shi et al. [22] investigated the machining of an ANSI 304 stainless steel workpiece. They reported that in the stationary condition of the tool electrode, the discharge phenomenon was not as prominent as compared to rotary tools.

The reported literature reveals that the hybrid MECDM process mainly focused on machining non-conductive materials. It also depicts that using a powder-mixed electrolyte gives a better result than the conventional MECDM process. In contrast, only a small amount of work on conductive materials was documented, and it was limited to the micro-ECDM method only. An in-depth study on the influence of the machining parameters of MECDM on a conductive material in the presence of a powder-mixed electrolyte is still not documented. The inclusion of non-conductive powders such as alumina, silicon carbide, and others helps to maintain a micro gap between the tool and the workpiece, allowing the gas film to develop at the tool’s tip. Moreover, it also helps to avoid the occurrence of short circuits, which are frequently observed in conventional MECDM processes, while machining conductive materials at irregular/or manual feed rates.

In this article, micro-drilling was carried out on conductive materials, i.e., maraging steel mostly used in the aviation industry, using the powder-mixed MECDM method (PMECDM), and the output response was compared with the conventional MECDM process in terms of MRR and surface quality, i.e., surface roughness, variation in hardness, and residual stress. Further, an in-depth study was carried out to understand the influence of machining parameters on the output responses and optimize the process parameters. 

## 2. Materials and Methods

### 2.1. Setup Configuration for Experiments

**Figure 2:**
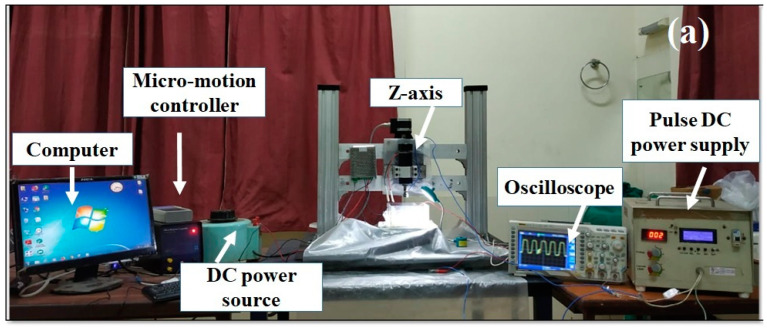

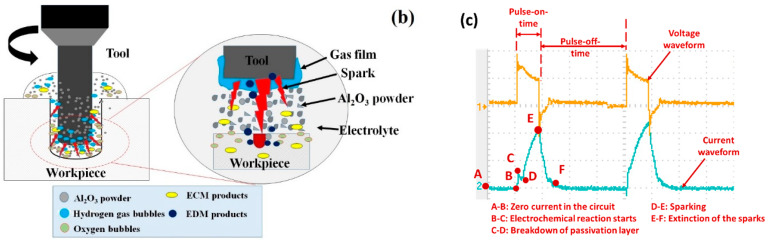
(**a**) Setup for the µ-ECDM; (**b**) Mechanism of material removal and spark formation in PMECDM process; (**c**) Voltage–current waveform during MECDM process.

An in-house designed and developed µ-ECDM setup is used to perform the experiments (Figure 2a). It consists of X-Y-Z CNC stages with a resolution of 0.125 μm. A micro-motion controller controls the motions and maintains the uniform tool/job feed motion. To perform the drilling operations, initially, the tool makes contact with workpiece and then retracts to 20 µm above the top surface of the workpiece and then maintains this gap throughout the drilling operation. A spindle with a rotational speed ranging from 100 to 1000 rpm is clamped on the Z-stage. The rotation of the tool helps to circulate the electrolyte even at the depth of the micro-hole. The electrolyte tank of dimension 100 × 100 × 60 mm^3^ is fabricated from the perspex sheet and facilitated with a work-holding device. A micro-submersible pump (3–6 V DC) circulates the electrolyte solution. DC pulse power supplied with an output voltage range: 10 V to 150 V, duty cycle: 5% to 70%, and frequency: 100 Hz to 10 kHz was used in the present context of study. In this research, the workpiece is conductive, hence it is made as anode. The tool electrode is connected to negative terminal of the power supply. An oscilloscope is attached to the power supply in order to capture the voltage–current waveform during the machining operations. The tool feed rate was decided after conducting several trial experiments [23].

### 2.2. Material Specifications

A commercially available tungsten rod with Ø500 µm was used as the cathode (tool), and the maraging steel sheet with dimensions 100 mm × 200 mm × 0.5 mm was used as workpiece. The maraging steel has the solubility of nickel–cobalt–molybdenum that leads to ultra-high-strength, good plasticity, and toughness without losing malleability [1]. The chemical composition (in wt%) of the maraging steel [24] is nickel: 17.9%, cobalt: 8.6%, molybdenum: 5.1%, titanium: 0.8%, aluminium: 0.1% and remainder is iron. It is extensively used in aviation, defense, and mold-making industries [24]. 

Two electrolytes were prepared (type 1: NaOH solution and type 2: NaOH + aluminum oxide powder-mixed (Al_2_O_3_) solution) to conduct two sets of experiments. In the second type, Al_2_O_3_ powder of average particle size 5 µm was mixed at 1% by weight ratio. Then ultra-sonication of the mixture was performed for 3 h to maintain the uniform dispersion of the particles. Based on the literature [25,26,27], three input parameters, namely voltage (V), duty cycle (D), and electrolyte concentration of the working solution (C), were considered for this study. 

### 2.3. Material Removal Mechanism

In the MECDM process of conductive materials, the DC pulse power supply creates electro-chemical sparks in the narrow gap between the tool and the workpiece. During the pulse-on-time, the electro-chemical reaction followed by the evolution of hydrogen gas occurs, and then the sparking phenomenon occurs. The typical current and voltage waveform captured during the machining process is presented in Figure 2c. A current probe was used for this purpose. There is no current in the electro-chemical cell corresponding to A–B section of Figure 2c. The electro-chemical reaction starts from point B till point C. During this path (B–C), passivation layer is built-up and at point C the breakdown of this layer takes place thereby, spark is established and exists until point D after which it disappears. This phenomenon is repeated in each cycle of the pulse power supply. However, for stable machining more pulse-off-time must be provided. 

The thermal energy liberated due to the sparks at tool leads to melting and vaporization of work material and forms a crater. The formed molten material is partially removed and remaining part sticks to the crater surface. Mixing abrasive/powder particles with the electrolyte increases the rubbing (grinding) action, impacting the workpiece intermittently from various angles. This grinding action helps eliminate the debris that sticks to the machined surfaces, and hence smooth and clean holes are obtained [17]. The abrasive particles are energized due to the shock wave generated during the sparks and the tool rotation. Figure 2b illustrates the mechanism of extraction of material and spark formation in Al_2_O_3_ mixed ECDM process. 

### 2.4. Design of Experiments

Several trial experiments were performed to find the suitable range of working parameters, i.e., voltage: 30 V to 50 V; duty factor (D): 20% to 30%; and electrolyte concentration (C): 10 to 20% wt./v. The other machining parameters such as frequency: 10 kHz, electrolyte level: 2 mm above the work surface, and tool rotation speed: 500 rpm are kept constant. Response surface methodology (RSM) was adopted to design the experiments in which the Box–Behnken design with 15 combinations of input parameters was chosen for both MECDM and PMECDM (indicated in Table 1) experiments (total run: 30). This methodology is capable of providing optimal results in a very limited number of experiments. This also aids in minimizing resource waste [28]. The measured values of the responses were entered in the response columns of the design Table 1 (obtained by using Minitab R14 software), and analysis of variance (ANOVA) was carried out for the development of regression equations and to examine its significance level. For all the analyses, 95% confidence levels were considered. 

### 2.5. Experimentation and Characterization Techniques

The experiments were conducted as per the design in Table 1. The workpiece and tool were prepared using wire-electrical discharge machine (WEDM). The workpiece was held in a vice placed in the machining tank, and tool was mounted on the spindle collet and rotated. The parameter settings were applied across them to drill the micro-holes. After the drilling operation, the prepared workpieces were cleaned thoroughly and dried post the machining operation. The weight-loss method was used to determine the amount of material removal rate and calculated using Equation (1) [29].

(1) $$\mathrm{MRR}=\frac{\left(W_{i}-W_{f}\right)}{t}$$

where ‘W_i_’ and ‘W_f_’ are the weight of the work material in milligrams before and after machining process, and “t” is the machining time. A digital weighing machine (Make: Mettler Toledo, model: MS205DU) was used for measuring weight. A non-contact type surface profiler (Made: Tayor Hobson, Model: Talysurf CCI HD M112-4449-01) was used for measuring the roughness on the periphery wall along the axis of the hole. A 3D surface profilometer (Zygo 9000) was used to capture the machined holes’ topographical images. Scanning Electron Microscopy (SEM: Zeiss EVOM 10) was used to observe the microstructure. Microhardness (Make: Matsuzawa Model: MMTX7) measurements were taken at a load of 50 gf using a diamond square pyramid indenter. Residual stress measurements were carried out using triaxial method by using a portable XRD machine from Proto Manufacturing Inc., LaSalle, ON, Canada, Model: iXRD. 

### 2.6. Regression Modelling 

The values of SR and the MRR for both processes were recorded and are presented in Table 1, and the respective values were entered in the design table of MINITAB software. ANOVA analysis was carried out to observe the effect of individual parameters, and their quadratic and interaction forms on different responses. The details of individual responses are described below. 

#### 2.6.1. ANOVA Analysis

Table 2, Table 3, Table 4 and Table 5 represent the ANOVA for material removal rate (MRR) and surface roughness (SR) in MECDM and PMECDM, respectively. The respective regression equation is presented in Equations (2)–(5). The ANOVA analysis was carried out to evaluate the statistical significance of the equations [30]. The high F-value in the ANOVA tables indicates a significant term. In all the tables, the R^2^ and adjusted R^2^ values are above 98% and the predicted R^2^ value is above 91%. This indicates adequacy of the developed RSM models to predict the responses within the selected range of parameters. The *p*-value > 0.05 for a term indicates the term does not have a significant contribution. The lack of fit value >0.05 (insignificant) indicates the good fitting of the model. As in Table 3 and Table 4, the *p*-value for the interaction is >0.05, and the interaction effects for all the combinations of parameters were plotted (Section 3).

#### 2.6.2. Regression Equations

The regression equations (Equations (2)–(5)) were developed to correlate the input process parameters and the measured responses. The fitness of the regression equation was checked with the help of ANOVA analysis (Section 2.6.1). Using these equations, the responses were examined for a set of input parameters (within the range, Table 2) and were plotted for further analysis. Table 2, Table 3, Table 4 and Table 5 show the ANOVA table for the regression Equations (2)–(5).

MRR_Conv_ = −0.149 − 0.0448V + 0.0534D + 0.0240C + 0.000339V × V − 0.002120D × D (2)

− 0.000483C × C + 0.001740V × D − 0.000291V × C + 0.001174D × C

SR_Conv_ = 5.579 − 0.2224 V + 0.0035 D − 0.0840 C + 0.003028 V × V − 0.000076 D × D (3)

+ 0.002176 C × C − 0.000404 V × D + 0.000094 V × C + 0.000764 D × C

MRR_PMECDM_ = 2.979 − 0.1663 V + 0.0456 D − 0.1453 C + 0.000845 V × V − 0.005877 D × D (4)

+ 0.001793 C × C + 0.005957 V × D − 0.000286 V × C + 0.005888 D × C

SR_PMECDM_ = 5.312 − 0.16916 V − 0.0564 D − 0.0976 C + 0.002061 V × V + 0.000636 D × D (5)

+ 0.002512 C × C + 0.000213 V × D + 0.000319 V × C + 0.000635 D × C

where MRR_Conv_: material removal rate in conventional MECDM, SR_Conv_: material removal rate in micro-ECDM, MRR_PMECDM_: material removal rate in PMECDM, SR_PMECDM_: surface roughness of PMECDM.

## 3. Result and Discussions

After conducting all the experiments, the prepared samples were cleaned thoroughly, and the characterization procedure was followed in a sequential manner. A total of 30 holes were drilled on the machined samples (15 for micro-ECDM and 15 for powder-mixed micro-ECDM). To analyze the micro-hole wall’s surface, the samples were sectioned using the WEDM process with a wire diameter of 150 microns. The characterization results were analyzed and presented in the succeeding sections to compare the performance of the MECDM and PMECDM processes. 

### 3.1. Effect of Input Parameters on MRR

**Figure 3:**
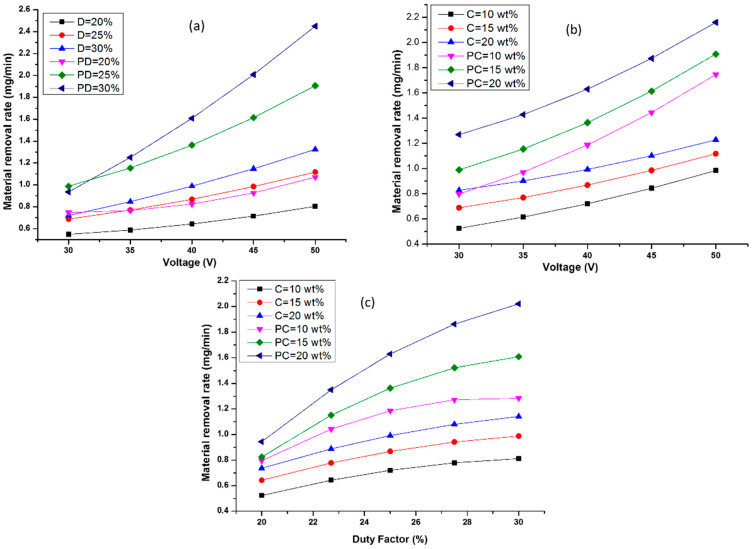
Impact of voltage (V) on MRR (**a**) at D and PD (**b**) at variable C and PC. Effect of duty factor on MRR (**c**) at various C and PC. Note: D: Duty factor, C: Concentration of working fluid at MECDM process and PD and PC are duty factor and concentration at PMECDM.

The MRR influences the efficiency of all machining processes. Using Equations (2) and (4), graphs (Figure 3a–c) were drawn to apprehend the effect of input machining factors on MRR in both the MECDM and PMECDM processes. The second process shows better MRR in comparison to the MECDM process. The presence of Al_2_O_3_ powder in PMECDM causes grinding action in addition to thermal melting and electro-chemical reactions [18,29,31]. During the discharge process, a shock wave is generated. The abrasive particles in close vicinity receive enormous kinetic energy and strike and rub the partially solidified debris/recast layer. This phenomenon imparts grinding action on the material surface and cleans most of the debris from the surface, hence the MRR increases in the PMECDM process as compared to the MECDM process. On a rise in voltage, the electrolyte concentration, duty factor, and the strength of the spark increases as the energy input to the process increases, therefore, the grinding action rises in addition to the material erosion due to the melting and electro-chemical reaction [18,32]. At a low voltage (30 V) and DF (20%), the MRR is 0.58 mg/min, which is improved by nearly 2.24 times at a high voltage (50 V) and a DF (30%) at 15 wt% of electrolyte concentration. A similar observation was reported by Varghese and Paul [33].

Similarly, increasing the DF and EC (at a constant machining voltage: 40 V) by 10% and 10 wt%, respectively (Figure 3c), improves the MRR by approximately 132%. By increasing the concentration, the mobility of ions increases, leading to enhancing the electro-chemical activity across the tool and forming bubbles of larger sizes which coalesce to develop a thick film. This results in high and intense spark energy [25,26]. Therefore, in all the plots (Figure 3a–c), the MRR shows an increasing trend for both machining approaches. 

### 3.2. Effect of Input Parameters on SR 

**Figure 4:**
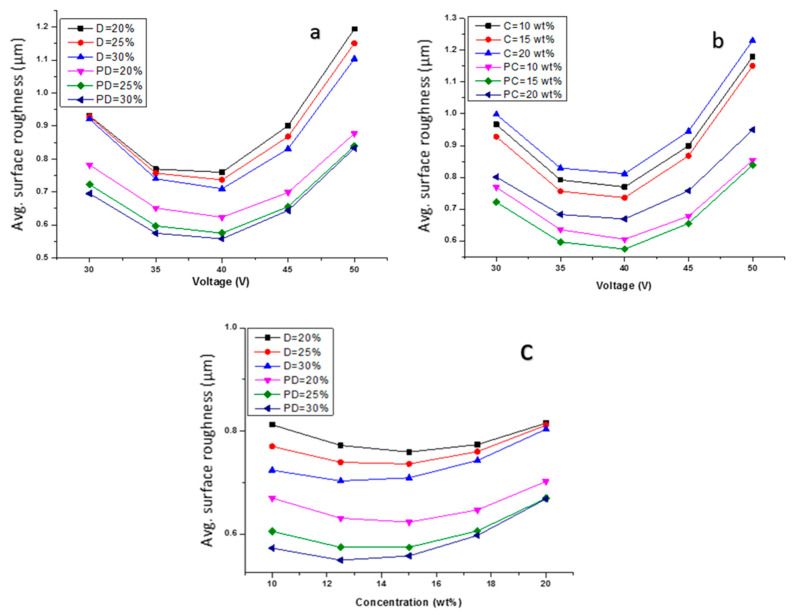

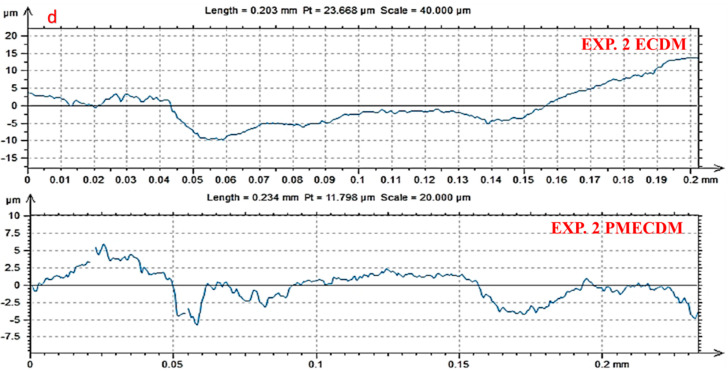
Effect of applied voltage on average surface roughness at different (**a**) duty factors, (**b**) voltages and (**c**) electrolyte concentrations, (**d**) surface profile of both the processes with same input parameters (D: duty factor for MECDM, PD: duty factor for PMECDM, C: electrolyte concentration for MECDM, PC: electrolyte concentration in PMECDM).

The average surface roughness (SR) is one of the important parameters for evaluating the quality of the surface. In this study, the regression Equations (3) and (5) were used for the construction of different plots presented in Figure 4a–c. In all the plots, the PMECDM produces surfaces with lower roughness as compared to the micro-ECDM process. This is because of the grinding action of the abrasive particles present in the electrolyte [17]. The details were discussed in the preceding section. Beyond 40 V of applied voltage, the SR increases in all the cases because of the generation of intense sparks and the flushing condition is not sufficient to remove the debris from the machining zone. Therefore, for a better MRR with a lower SR, the input parameters are to be selected carefully. The surface roughness achieved by PMECDM is lower than that of the MECDM process.

### 3.3. Surface Topography

**Figure 5:**
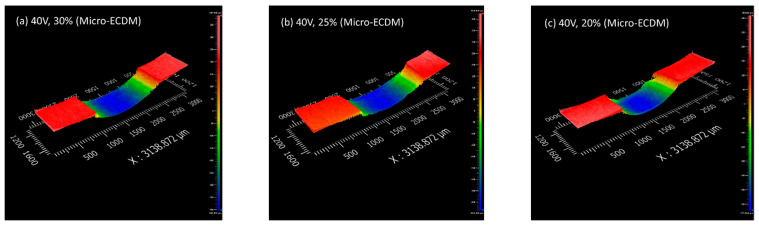

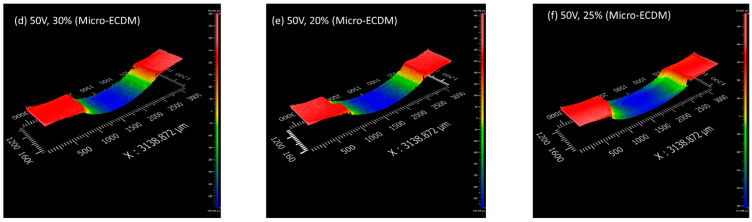
Three-dimensional surface profiles of the holes prepared using MECDM.

**Figure 6:**
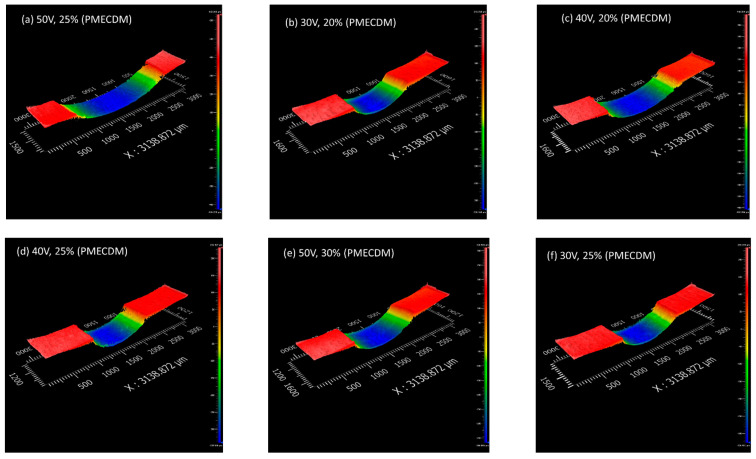
Three-dimensional surface profiles of holes prepared using PMECDM.

All the samples containing holes were sectioned and polished to observe the topography. Figure 5 and Figure 6 show a few of the captured images for MECDM and PMECDM at different machining parameters, respectively. The reddish region indicates the parent material, while the other colored regions show the sectioned hole’s surface. As the hole’s surface is curved, the different colors distinguish different parts of the hole’s inner wall. The irregular surfaces are observed in the case of MECDM, which is due to the deposition of debris. Due to the grinding action of the abrasive powder particles, the surfaces are clean in the case of PMECDM.

However, in the case of the PMECDM method, the entrance hole diameter was large as compared to the MECDM process. This is mainly because of side sparks that occurred due to the availability of the electrolyte at the narrow side gap maintained by the abrasive powder during the machining of the micro-hole [16]. However, in both cases, it was observed that the circularity was similar. 

### 3.4. Microstructure

The sectioned samples were examined in FESEM to check the presence of debris or any other defects on the hole wall. A clean surface was observed in the case of PMECDM as compared to the MECDM process. This is due to the induced grinding action by the abrasive particles during the discharge process as discussed in the above sections.

**Figure 7:**
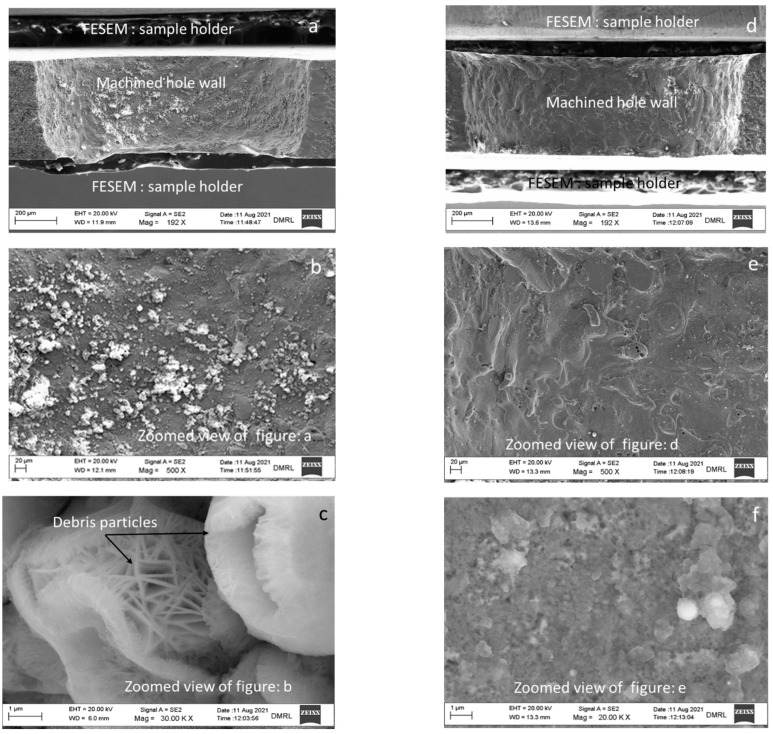
FESEM images of hole wall fabricated by (**a**–**c**) MECDM process and (**d**–**f**) PMECDM process (parameter settings: Applied voltage: 40 V, duty factor: 20%, electrolyte concentration: 20 wt%).

During machining, the spark is produced for a short duration of time, which leads to the formation of shock waves. The Al_2_O_3_ particles mixed in the electrolyte become energized, produce a rubbing action on the surface, and remove the debris (Figure 7b). This grinding phenomenon is absent in the micro-ECDM process, leading to debris accumulation (Figure 7a–c). Figure 7d–f show the images of hole surfaces processed through PMECDM. The grinding action may provide the work hardening action or incorporation of residual stress on the hole wall, the details of which are described in the next section.

### 3.5. Microhardness

**Figure 8:**
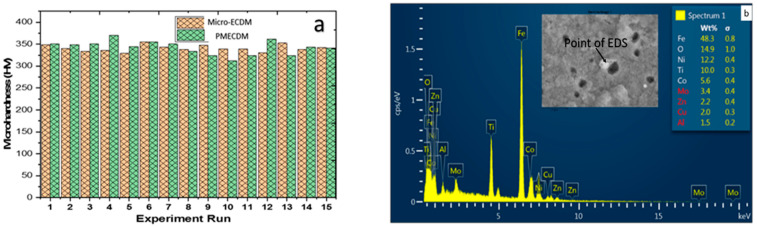
(**a**) Microhardness profile (**b**) point EDS of debris particle in PMECDM.

The average microhardness on the wall of the machined holes was measured and compared for both processes (Figure 8a). The hardness of the parent workpiece is 320 HV.

For measuring the microhardness of the machined surface, a standard Vickers hardness tester (model: Economet VH-1 MD, Make: Chennai Metco, Chennai, India, measuring range: 8–2900 HV) was used. Initially, the sample was cut into single holes through wire EDM. Then the sample was molded using a molding machine in order to properly clean and polish the top surface of the hole through multiple grades of polishing paper ranging from 600 to 2000 grit size. At last, indentation was carried out on the edge of the micro-hole at a gap of 50 µm from the hole edge. The 100 gf load was applied at a dwell time of 10 s. A total of five readings were taken across the edge and the average was considered for the analysis.

No significant change in microhardness was observed, which indicates the mixing of powder into the dielectric helps in debris removal but does not contribute to the change of hardness of the surface either by work hardening action or by forming a hard phase with the parent material. The point EDS result (Figure 8b) confirms the presence of Al in very low proportions on the surface of the hole wall which, at some places, a hard intermetallic compound may have formed and the microhardness value goes a little higher in the case of powder-mixed micro-ECDM. 

### 3.6. Residual Stress Analysis

**Figure 9:**
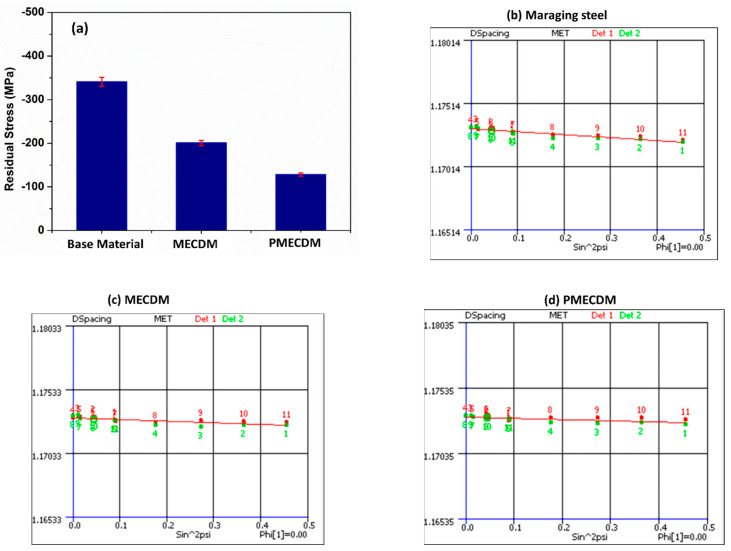
Comparison of residual stresses for different samples.

In the case of the electrical spark machining process, it is reported that tensile residual stresses are induced in the generated surface due to the rapid cooling of the molten metal pool after each spark. Due to this, tensile residual stresses are induced and when they exceed the fracture strength of the material, a crack is formed and may propagate to cause the permanent failure of the component. Therefore, exploring the type of stresses induced on the machined surface obtained through the spark erosion process is required [34]. In the present study, residual stress measurement was carried out using the X-ray diffraction (XRD) technique for the parent material, and samples were prepared using MECDM and PMECDM. Three samples from each category were taken for measurement, and the average was considered for further analysis. A Chromium rod (Cr) was used as the cathode and Tungsten as the anode. Stress was determined by the sin^2^ψ method by using in-built XRDWIN 2.0 software. XRD measurements are made at different Psi tilts. The inter-planar spacing or 2-theta peak position is measured and plotted as a curve; typical plots for each sample are shown in Figure 9b–d (at ψ = 0°). The standard used for this measurement is as per ASTM E 2860.

The periodic arrangement of atoms leads to the formation of a crystal structure; due to residual stresses, the position of atoms in the crystal structure is disturbed with respect to the ideal crystal structure of the material [35]. In XRD tests, these are identified through diffraction peaks. Many useful properties such as residual stress, crystal size, lattice strain, etc. can be quantified by analyzing these peaks. It was observed that the equivalent stress for the parent material is highest at −341.04 ± 10.24 MPa, whereas the samples prepared using the MECDM possess −200.7 ± 6.02 MPa and the PMECDM samples possess the lowest residual stress value of −128.3 ± 3.85 MPa (Figure 9a). In the ECDM process, the recast layer is removed by an electro-chemical reaction due to which the residual stress becomes negative (compressive). The results also indicate that due to the mixing of powders (Figure 9d), the residual stress does not become tensile in nature. Therefore, by using a powder-mixed electrolyte, the compressive residual stresses in the hole surface are further reduced but never attained tensile values. The efficient removal of debris is desirable in the case of micro-hole drilling so it is recommended to use a powder-mixed electrolyte. 

## 4. Conclusions

Micro-hole drilling experiments were conducted using an exotic material, i.e., maraging steel through hybrid MECDM methods to fulfill the requirement of the aviation industry. The paper also compared the machining performances of conventional MECDM and PMECDM processes. It described the novel approach of machining an exotic conductive material with high material removal rate simultaneously with a high surface finish. A laboratory-scale µ-ECDM setup was used for this purpose. The first set of experiments was conducted using a NaOH (aq.) electrolyte. The second set was conducted with a Al_2_O_3_ powder-mixed NaOH (aq.) electrolyte. The outcome of the characterization states the following conclusions:

1. The hybridization of the MECDM process can be performed easily by mixing powder additive(s) with the electrolyte, which makes the process more efficient without any modification to the setup.
2. High MRR, low average surface roughness, and minimum accumulation of debris on the hole wall was observed in the PMECDM process.
3. In the PMECDM process, the highest MRR achieved was 2.44 mg/min with input parameters of 50V, duty factor: 30% and electrolyte concentration: 15 wt% whereas the lowest average surface roughness was obtained with machining parameters of 40V, duty factor: 25% and electrolyte concentration: 15%.
4. The mixing of 1 wt% Al_2_O_3_ powder to the NaOH (aq.) electrolyte in the PMECDM process leads to an average increase in MRR by 34% and a reduction in average surface roughness by 21% as compared to the MECDM process.
5. The grinding action of the abrasives in PMECDM helps enhance the MRR and minimize the SR without compromising the other surface properties.
6. The holes fabricated with the PMECDM process had the lowest residual stress (−128.3 ± 3.85 MPa) as compared to MECDM (−200.7 ± 6.02 MPa) and the base material (−341.04 ± 10.24 MPa), which is beneficial for the service life of the component. However, further improvement to the surface integrity can be carried out by fine-tuning the experimental process parameters and by altering the particle size, concentration and type of powders.

## Figures and Tables

**Table 1:** Parameter settings of experimental runs and responses.

Exp. No.	Voltage (V)	Duty Cycle (D)	Concentration (% wt.)	Micro-ECDM		PMECDM	
				MRR (mg/min)	SR (µm)	MRR (mg/min)	SR (µm)
1	40	25	15	0.853	0.737	1.339	0.567
2	50	25	20	1.236	1.240	2.160	0.965
3	40	30	20	1.168	0.793	2.019	0.670
4	40	20	10	0.502	0.819	0.791	0.670
5	40	25	15	0.877	0.723	1.360	0.581
6	50	25	10	0.997	1.161	1.706	0.856
7	40	20	20	0.719	0.793	0.898	0.690
8	50	30	15	1.297	1.097	2.438	0.820
9	30	25	20	0.817	1.012	1.301	0.801
10	50	20	15	0.819	1.198	1.104	0.877
11	30	20	15	0.580	0.933	0.752	0.797
12	30	25	10	0.520	0.952	0.789	0.756
13	30	30	15	0.710	0.913	0.895	0.697
14	40	30	10	0.833	0.742	1.323	0.587
15	40	25	15	0.882	0.743	1.380	0.579

SR: Average Surface Roughness.

**Table 2:** ANOVA for MRR in MECDM.

Source	DF	Adj SS	Adj MS	F-Value	*p*-Value
Model	9	0.8092	0.8991	94.33	0.000
Linear	3	0.7585	0.2528	265.24	0.000
Square	3	0.0162	0.0054	5.66	0.046
2-way interaction	3	0.0346	0.0115	12.09	0.010
Error	5	0.0047	0.0009		
Lack-of-fit	3	0.0043	0.0014	6.17	0.143
Pure error	2	0.0004	0.00023		
Total	14	0.8139			

R^2^: 99.41%; R^2^ (Adj): 98.36%; R^2^ (Pred): 91.42%.

**Table 3:** ANOVA for MRR in PMECDM.

Source	DF	Adj SS	Adj MS	F-Value	*p*-Value
Model	9	3.8665	0.4296	221.61	0.000
Linear	3	3.3014	1.1005	567.67	0.000
Square	3	0.1228	0.0409	21.12	0.003
2-way interaction	3	0.4423	0.14743	76.05	0.000
Error	5	0.0096	0.00194		
Lack-of-fit	3	0.0088	0.00296	7.15	0.125
Pure error	2	0.0008	0.00041		
Total	14	3.8763			

R^2^: 99.75%; R^2^ (Adj): 99.30%; R^2^ (Pred): 96.29%.

**Table 4:** ANOVA for SR in MECDM.

Source	DF	Adj SS	Adj MS	F-Value	*p*-Value
Model	9	0.4544	0.0504	118.49	0.000
Linear	3	0.1063	0.0354	83.21	0.000
Square	3	0.2448	0.1149	269.77	0.000
2-way interaction	3	0.0031	0.0010	2.49	0.175
Error	5	0.0021	0.0004	6.30	
Lack-of-fit	3	0.0019	0.0006		0.140
Pure error	2	0.0002	0.0001		
Total	14	0.4565			

R^2^: 99.53%; R^2^ (Adj): 98.69%; R^2^ (Pred): 93.15%.

**Table 5:** ANOVA for SR in PMECDM.

Source	DF	Adj SS	Adj MS	F-Value	*p*-Value
Model	9	0.2115	0.0235	93.26	0.000
Linear	3	0.0439	0.0146	58.16	0.000
Square	3	0.1650	0.0550	218.34	0.000
2-way interaction	3	0.0024	0.0008	3.28	0.117
Error	5	0.0012	0.0002		
Lack-of-fit	3	0.0011	0.0003	6.66	0.133
Pure error	2	0.0001	0.0000		
Total	14	0.2127			

R^2^: 99.41%; R^2^ (Adj): 98.34%; R^2^ (Pred): 91.27%.

## Data Availability

The data are available from the corresponding author upon request.
